# Forecasting daily emergency department arrivals using high-dimensional multivariate data: a feature selection approach

**DOI:** 10.1186/s12911-022-01878-7

**Published:** 2022-05-17

**Authors:** Jalmari Tuominen, Francesco Lomio, Niku Oksala, Ari Palomäki, Jaakko Peltonen, Heikki Huttunen, Antti Roine

**Affiliations:** 1 Faculty of Medicine and Health Technology, Tampere University, Arvo Ylpön katu 34, 33520 Tampere, Finland; 2 Faculty of Information Technology and Communication Sciences, Tampere University, Tampere, Finland; 3 Emergency Department, Kanta-Häme Central Hospital, Ahvenistontie 20, 13530 Hämeenlinna, Finland; 4 Vascular Centre, Tampere University Hospital, Elämänaukio, Kuntokatu 2, 33520 Tampere, Finland

**Keywords:** Emergency department, Crowding, Feature selection, Machine learning, Time series forecasting, Statistical learning

## Abstract

**Background and objective:**

Emergency Department (ED) overcrowding is a chronic international issue that is associated with adverse treatment outcomes. Accurate forecasts of future service demand would enable intelligent resource allocation that could alleviate the problem. There has been continued academic interest in ED forecasting but the number of used explanatory variables has been low, limited mainly to calendar and weather variables. In this study we investigate whether predictive accuracy of next day arrivals could be enhanced using high number of potentially relevant explanatory variables and document two feature selection processes that aim to identify which subset of variables is associated with number of next day arrivals. Performance of such predictions over longer horizons is also shown.

**Methods:**

We extracted numbers of total daily arrivals from Tampere University Hospital ED between the time period of June 1, 2015 and June 19, 2019. 158 potential explanatory variables were collected from multiple data sources consisting not only of weather and calendar variables but also an extensive list of local public events, numbers of website visits to two hospital domains, numbers of available hospital beds in 33 local hospitals or health centres and Google trends searches for the ED. We used two feature selection processes: Simulated Annealing (SA) and Floating Search (FS) with Recursive Least Squares (RLS) and Least Mean Squares (LMS). Performance of these approaches was compared against autoregressive integrated moving average (ARIMA), regression with ARIMA errors (ARIMAX) and Random Forest (RF). Mean Absolute Percentage Error (MAPE) was used as the main error metric.

**Results:**

Calendar variables, load of secondary care facilities and local public events were dominant in the identified predictive features. RLS-SA and RLS-FA provided slightly better accuracy compared ARIMA. ARIMAX was the most accurate model but the difference between RLS-SA and RLS-FA was not statistically significant.

**Conclusions:**

Our study provides new insight into potential underlying factors associated with number of next day presentations. It also suggests that predictive accuracy of next day arrivals can be increased using high-dimensional feature selection approach when compared to both univariate and nonfiltered high-dimensional approach. Performance over multiple horizons was similar with a gradual decline for longer horizons. However, outperforming ARIMAX remains a challenge when working with daily data. Future work should focus on enhancing the feature selection mechanism, investigating its applicability to other domains and in identifying other potentially relevant explanatory variables.

**Supplementary Information:**

The online version contains supplementary material available at 10.1186/s12911-022-01878-7.

## Introduction

Emergency Departments (ED) worldwide serve a crucial purpose, providing immediate care to patients presenting with health conditions that vary from minor to life-threatening. In this setting, the ability to provide timely and high-quality care is of utmost importance. Unfortunately, ED’s all over the world suffer from regular overcrowding which has been repeatedly associated with suboptimal care leading to both increased morbidity [1] and increased 10 days mortality [2–4]. The ability to successfully forecast future overcrowding would enable better resource allocation that could alleviate the problem or even eliminate it altogether.

Following this rationale, there has been a continued academic interest in ED forecasting [5] but much of the previous work has focused on investigating applicability of different algorithms [6–9] or the predictive value of a singular independent variable such as website visits [10], road traffic flow [11] or aggregated acuity of admitted patients [12]. Due to extremely interdependent nature of ED’s the number of potential input features is high and testing each of them one by one is a painstaking process. Moreover, since these input features likely demonstrate significant multicollinearity, testing them one by one can provide a misleading picture of their relative importance. Despite these issues, there has been little to no emphasis on the number and quality of the used independent variables and, most importantly, on their aggregated value when used in conjunction with one another.

Reluctancy towards high-dimensional multivariate input is understandable from both computational and practical standpoint. From computational perspective the amount of added noise is usually proportional to number of input dimensions which often leads to loss of predictive accuracy. Moreover, ED forecasting is almost always performed using statistical time series forecasting algorithms [5] most of which are strictly univariate by design, with the notable exception of regression with ARIMA errors (ARIMAX). It is thus not a coincidence that ARIMAX with very limited and arbitrarily selected calendar and weather variables seems to outperform other statistical models [12, 13]. We hypothesise, that if this kind of arbitrary feature selection works as well as it does, it should be possible to completely automate the feature selection process, which would make it significantly faster to identify useful input features and potentially enhance model accuracy.

Feature selection processes have conventionally been utilized in pre-processing of imaging and biomedical signals as well as in genetic studies. In addition to eliminating noise and increasing computational speed, they can provide new understanding on the factors behind the phenomenon of interest [14] which could ultimately inform wider health care policies. To our knowledge there is only one publication by Jiang et al. that has documented a feature selection process specifically in the ED forecasting context. However, even then the selection is done out of a very limited set of weather and holiday variables, which questions the necessity and performance of their approach [15].

In this empirical study we demonstrate a feature selection process to identify predictors of ED crowding using a dataset from a large Nordic ED along with a largest-to-date collection of predictor candidates. Using this data, we test two feature selection mechanisms: simulated annealing and floating search and benchmark our results against current gold standard.

## Materials and methods

### Data

Tampere University Hospital is an academic hospital located in Tampere, Finland serving a population of 535,000 in Pirkanmaa hospital district and as a tertiary hospital an additional population of 365,700 and providing level 1 trauma center equivalent capabilities. The hospital ED *“Acuta”* is a combined ED with total capacity of 111–118 patients with 70 beds (and additional 7 beds as a reserve) and 41 seats for walk-in patients. Approximately 100,000 patients are treated annually. For this study, the daily numbers of all registered ED visits were obtained from hospital database created during the sample period from June 1, 2015 to June 19, 2019 resulting in 386 579 individual visits. The number of next day total arrivals (DTA) was used as the target variable.

Based on previous literature and intuition, explanatory variables were collected from different data sources as listed in Table 1. Historical weather data was acquired in hourly resolution from the nearest observation station [16]. Timestamps of Finnish holidays were provided by University Almanac Office [17]. Calendar variables were encoded according to their status as national holidays and working days. Additionally, we included each national holiday as a categorical variable since their impact on ED service demand likely differs significantly due to different levels of social activity. Weekdays and months were also included as can be expected.

**Table 1:** List of potential explanatory variables

Variable name	N columns	Type	Lag (days)
N of available hospital beds	33	Int	− 1
N of available hospital beds	1	Float	− 1
N of available hospital beds_Σ_	1	Float	− 1
Weekday	7	Binary	0
Month	12	Binary	0
Specific holiday	18	Binary	0
Lagged holiday	3	Binary	0
Working day	1	Binary	0
Cloud count	1	Int	0
Air pressure	1	Float	0
Relative humidity	1	Float	0
Rain intensity	1	Float	0
Snow depth	1	Float	0
Air temperature	1	Float	0
Dew point temperature	1	Float	0
Visibility	1	Int	0
Air temperature min	1	Float	0
Air temperature max	1	Float	0
Website Visits_tays.fi_	1	Int	− 1
Website Visits_tays.fi/acuta_	1	Int	− 1
Ekstöm's visits_tays.fi_	1	Int	− 1
Ekström's ratio_tays.fi_	1	Int	− 1
Google Trends_"Acuta"_	1	Int	− 1
N of minor public events	1	Int	0
N of major public events	1	Int	0
N of all public events	1	Int	0
Specific public event	65	Binary	0
	158		

*N* number, *Int* integer, *float* floating point, *N Columns* number of columns

Timestamps of local public events were provided by Tampere city officials. The provided log contained an event name, date of organisation and event size. Two feature sets were engineered using this data. First, we computed a timeseries of the total number of ongoing events each day within the Tampere area, with the hypothesis that increased activity (and often increased substance consumption) might have an impact in ED service demand. The total number of events was further divided by event size into the number of minor and major public events. Additionally, we identified 73 recurring events that are organized each year. These events contained a wide array of social activities from concerts to sports events. The events were included as individual binary vectors, since, analogous to different holidays, different events likely have different, or even contradicting, impact on service demand.

**Fig. 1:**
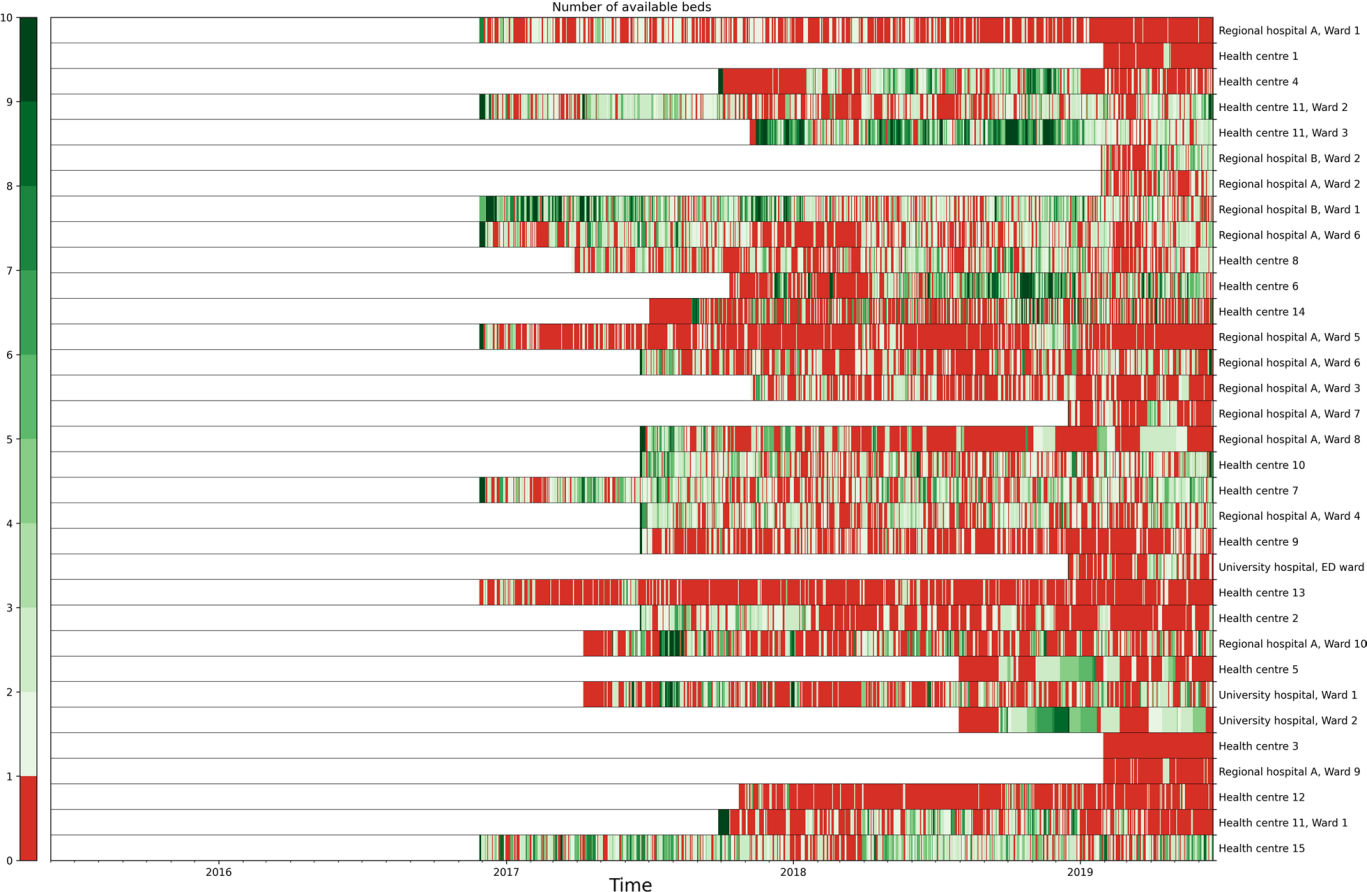
Temporal availability of beds in 33 catchment area hospitals or health centres as extracted from Uoma© which is a software developed by Unitary Healthcare Ltd. used to facilitate easier patient transfers. Negative availability is drawn as 0 for clarity. White space represents missing data, caused mainly by sequential introduction of the software. There are interesting differences between facilities, some demonstrating constant overload which likely significantly contributes to catchment area access block

A timeseries containing the number of available beds in 34 inpatient facilities in Pirkanmaa Hospital district catchment area was provided by Unitary Healthcare Ltd which provides a logistics software for patient transfers. The rationale of including these features into the dataset resides in the hypothesis that the availability of hospital beds is inversely correlated with ED arrivals. More precisely, if a primary care physician is unable to find a bed for a patient in need, they are often forced to send the patient to the ED merely to organise the bed that the patient requires. In addition to including the capacity of each individual hospital and health care centre we also included both the mean and sum of all the available beds on any given day. Temporal availability of hospital beds in included facilities is visualised in Fig. 1.

The numbers of website visits to two domains (www.tays.fi and www.tays.fi/acuta) were acquired from Tampere University Hospital Information Management. The former of these was available in hourly resolution and the latter in daily resolution. Daily sums of visits to both domains were included. Additionally, we summed the visits between 18 pm and midnight in the identical manner as was suggested and justified by Ekström et al. and named this feature as “*Ekström’s visits*” [10]. Moreover, a stationary version of this variable was included by dividing the evening visits by earlier visits during the day. This variable is referred to as “*Ekström’s ratio*”. The number of daily Google searches for word “Acuta” was also used as an input [18].

Website visits, Google searches and available hospital beds were lagged by one day whereas weather variables were not, assuming that weather can be forecasted with satisfying precision one day ahead. All explanatory variables are collected and presented in Table 1.

### Models

#### Benchmark models

Autoregressive Integrated Moving Average (ARIMA) is a widely used statistical forecasting model the performance of which has been previously extensively documented in ED forecasting [5, 19]. It has established a position as one of the most important benchmarks not only in ED forecasting but in time series forecasting in general [12, 20]. Due to established nature of the model, we refer to Chapter 9 of [21] for the basic concepts. In essence, ARIMA is a combination of three components: autoregression (AR), integration (I) and moving average (MA). Integration step serves to ensure stationarity of the data. Number of required differences and the length of history that is used as an input for AR and MA components constitutes the model order which is referred to as (*p*, d, q) in which *p* is the number of time lags for AR, d is number of differencing and q is number of time lags for MA. The order of the model is then determined either manually by dedicated statistical procedures or using an automated approach. When additional independent variables are used in conjunction with the univariate historical signal, the model is referred to as regression with ARIMA errors or ARIMAX. For seasonal data, it is often useful to define time lags as a multiple of the known seasonality and perform seasonal differencing, in which case the model is referred to as Seasonal ARIMA or SARIMA. In this study, model order was defined with Auto-ARIMA as initially described by Hyndman et al. [22] using a Python implementation provided by Smith et al. [23]. Auto-ARIMA is a relatively complex algorithmic approach which completely automates both the order and parameter estimation of ARIMA modelling. This is ideal for potential future implementation since manual order estimation would be very laborious if done hourly or daily. The code used to produce ARIMAX results is provided in Additional File 2. Using this approach we provide three ARIMA benchmarks: one trained with both univariate signal and all 158 explanatory variables (ARIMAX-A), one trained only with univariate historical signal (ARIMA) and one trained with features inspired by work of Whitt et al. [13] (ARIMAX-W) containing a limited number of weather and calendar variables. ARIMAX trained with features identified by simulated annealing and floating search are referred to as ARIMAX-SA and ARIMAX-FS respectively. The known weekly seasonality of the target variable was provided to the optimizer which automatically defines whether seasonal lags are required for best available fit.

We also include Random Forest (RF) as a benchmark, which is one of the most used machine learning models and is particularly beneficial in the case of high dimensional data since it natively uses subsets of the input data. In addition, it can work well with features of different types (binary, numerical, categorical). It is an ensemble technique, meaning that it uses a set of simpler models to solve the assigned task [24]. In this case, RF uses an ensemble of decision trees. An arbitrary number of decision trees is generated, each considering a randomly chosen subset of the samples of the original dataset. To reduce the correlation between the individual decision trees, a random subset of the features of the original dataset is selected. The hyperparameters of the RF were selected using a randomized search algorithm [25]. More specifically, the search was made among the *number of estimators*, the depth of the tree and whether it uses or not the bootstrap. The best RF uses 1000 estimators, uses bootstrap and has no limit on the depth. Each tree is therefore trained on its subset of the data, and it can give a prediction on new unseen data. The RF regressor uses the results of all these trees and averages them to generate the prediction. Four versions of RF with different inputs were tested: RF-U with only univariate signal, RF-FS with variables identified by FS, RF-SA with variables identified with SA and RF-A with all variables.

Naïve and Seasonal Naïve (SNaive) were also included as benchmark models to establish the ultimate baseline of performance. Naïve model uses the latest observed value as the prediction, e.g. when predicting arrivals of Wednesday, observed values of Tuesday are used. SNaive uses the latest observed value a season ago as the prediction, e.g. when predicting arrivals of next Wednesday, observed value of last Wednesday is used.

#### LMS and RLS filters

Due to the nature of the data used, characterized by seasonal variations and high number of input dimensions, we focused our attention on classical signal processing including LMS filters and RLS filters [26]. These models have the benefit of being both simple and efficient which is required due to high number of train-test iterations in the feature selection phase. LMS and RLS can be characterized as gradient learning models, as they adjust the model parameters according to the gradient of the prediction error.

LMS filter is a digital Finite Impulse Response filter with time-varying (adaptive) weights. As such the LMS filter is commonly used for adaptive signal processing tasks, where the environment changes dynamically such as echo cancellation [26]. As the environment in our study is not necessarily stationary, and all latent factors affecting the dynamics are not measurable, the prediction model needs to be able to adapt to the changes in the input–output relationships and the LMS filter is able to do so.

The LMS filter can be formulated as follows. Denote the prediction target (e.g. ED arrivals) at time step n as y(n), and inputs as **x**(n), n = 1,2,…,N. The inputs are constructed as a vector, whose elements in our case consist of both endogenous variables (historical values of arrivals) and explanatory variables. The LMS filter predicts the output $\hat{y}\left( n \right)$ as a weighted sum (inner product) of inputs and weights:

$$\hat{y}\left( n \right) = {\varvec{h}}\left( n \right)^{T} {\varvec{x}}\left( n \right)$$

The weight vector **h**(n) is initialized with zeros and adaptively updated. The update computes the prediction error $e\left( n \right) = y\left( n \right) - \hat{y}\left( n \right)$ and applies the gradient update rule:

$${\varvec{h}}\left( {n + 1} \right) = {\varvec{h}}\left( n \right) + \mu e\left( n \right){\varvec{x}}\left( n \right)$$

where $\mu > 0$ is the learning rate.

The Recursive Least Squares (RLS) filter is another adaptive filtering formulation, that has significantly faster convergence compared to LMS. The RLS filter is approximate the theoretical solution for the weight vector **w** minimizing the prediction error:

$${\varvec{w}}\left( {\varvec{n}} \right) = {\varvec{R}}^{ - 1} \left( {\varvec{n}} \right){\varvec{r}}\left( {\varvec{n}} \right),$$

where **R** is the expectation of the autocorrelation matrix of input x, and **r** is the expectation of the cross-correlation of input x and target y:

$${\varvec{R}}\left( {\varvec{n}} \right) = \mathop \sum \limits_{{{\varvec{i}} = 0}}^{{\varvec{n}}} {\varvec{\lambda}}^{{{\varvec{n}} - {\varvec{i}}}} {\varvec{x}}\left( {\varvec{i}} \right){\varvec{x}}^{{\varvec{T}}} \left( {\varvec{i}} \right),$$

$${\varvec{r}}\left( {\varvec{n}} \right) = \mathop \sum \limits_{{{\varvec{i}} = 0}}^{{\varvec{n}}} {\varvec{\lambda}}^{{{\varvec{n}} - {\varvec{i}}}} {\varvec{y}}\left( {\varvec{i}} \right){\varvec{x}}\left( {\varvec{i}} \right),$$

Under a nonstationary situation, these correlations must be computed for each time step. In practical implementation, the expectations are replaced by their sample-based estimates which are updated at each time step to minimize a weighted prediction error that downweighs older errors. Moreover, the RLS algorithm directly updates the inverse of the autocorrelation matrix in order to avoid matrix inversion. Similar to the learning rate of the LMS filter, the speed of adaptation of the RLS filter can be controlled by the forgetting factor λ, which determines the weight given to old measurements.

### Feature selection

To obtain the most important features in terms of predictive accuracy, we used two different techniques: simulated annealing (SA) and floating search (FS). These algorithms were chosen since they are both fast to deploy and easy to understand. Moreover, both provide a faster execution compared to other greedy feature selection techniques, while still maintaining excellence performance.

SA consists of selecting an arbitrary variable and randomly selecting a neighbor to minimize the internal energy of the system. More specifically: for each variable selected, the algorithm selects a second and checks whether the new “solution” is better (low energy state) or worse than the previous one. If the selected feature improves the overall result, it is kept, otherwise a new variable is tested.

FS feature selection, iteratively adds and removes some of the variables until it reaches a stable subset of features. During the addition phase, the algorithm tests recursively one feature at the time, adding a new feature if this improves the result: this is done until 10 features are added. In the removal phase, it removes one feature at the times from the subsect selected in the previous phase, until the 5 least beneficial features are removed. The FS continues until it doesn’t exist a set of 10 features which improves the result when added, nor it exists a set of 5 features which improve when removed.

Both LMS and RLS were used as predictive models in feature selection phase, resulting in four models which are later referred to as LMS-FS, LMS-SA, RLS-FS and RLS-SA.

### Cross validation, error measures and statistical tests

The dataset was divided into training set containing the samples from June 1, 2015 to December 31, 2017 (944 days, 64%) and test set containing the samples from January 1, 2018 to June 19, 2019 (534 days, 36%). Out-of-sample accuracies over the test set were calculated using a rolling forecast origin with predictive horizon of one day. Mean Absolute Percentage Error (MAPE) was used as the error metric since it is scale-invariant and because its wide adoption allows comparisons to previous studies [5]. The formula for MAPE is defined as follows:

$${\text{MAPE}} = \frac{100}{n}\mathop \sum \limits_{i = 1}^{n} \frac{{\left| {y_{i} - \hat{y}_{i} } \right|}}{{y_{i} }}$$

where *n* = number of samples, $y_{i}$ = ground truth, $\hat{y}_{i}$ = prediction.

We used ANOVA and two-tailed Dunnett’s post-hoc test to investigate statistical significance between reported MAPE’s. Multiple comparisons to both Seasonal Naïve and to the best performing model were performed. Statistical significance was specified as *P* < 0.05. Statistical analyses were performed using SPSS Statistics version 27.0.1.0.

## Results

### Model accuracy

**Fig. 2:**
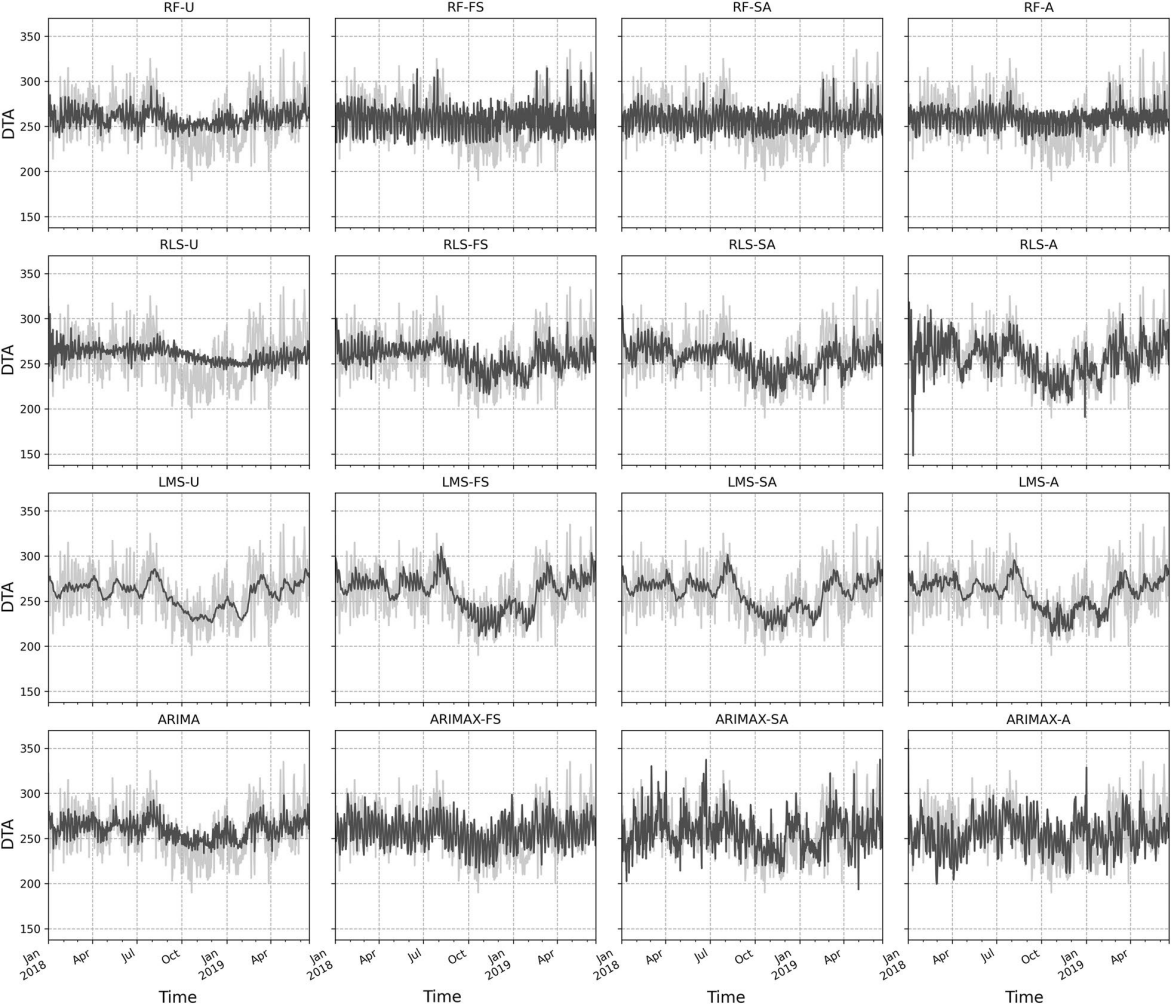
Predictions superimposed with ground truth. Light grey line = ground truth, dark grey line = prediction. RF = random forest, RLS = recursive least squares, LMS = least mean squares, ARIMA = autoregressive integrated moving average, ARIMAX = regression with ARIMA errors, FS = floating search, SA = simulated annealing

**Fig. 3:**
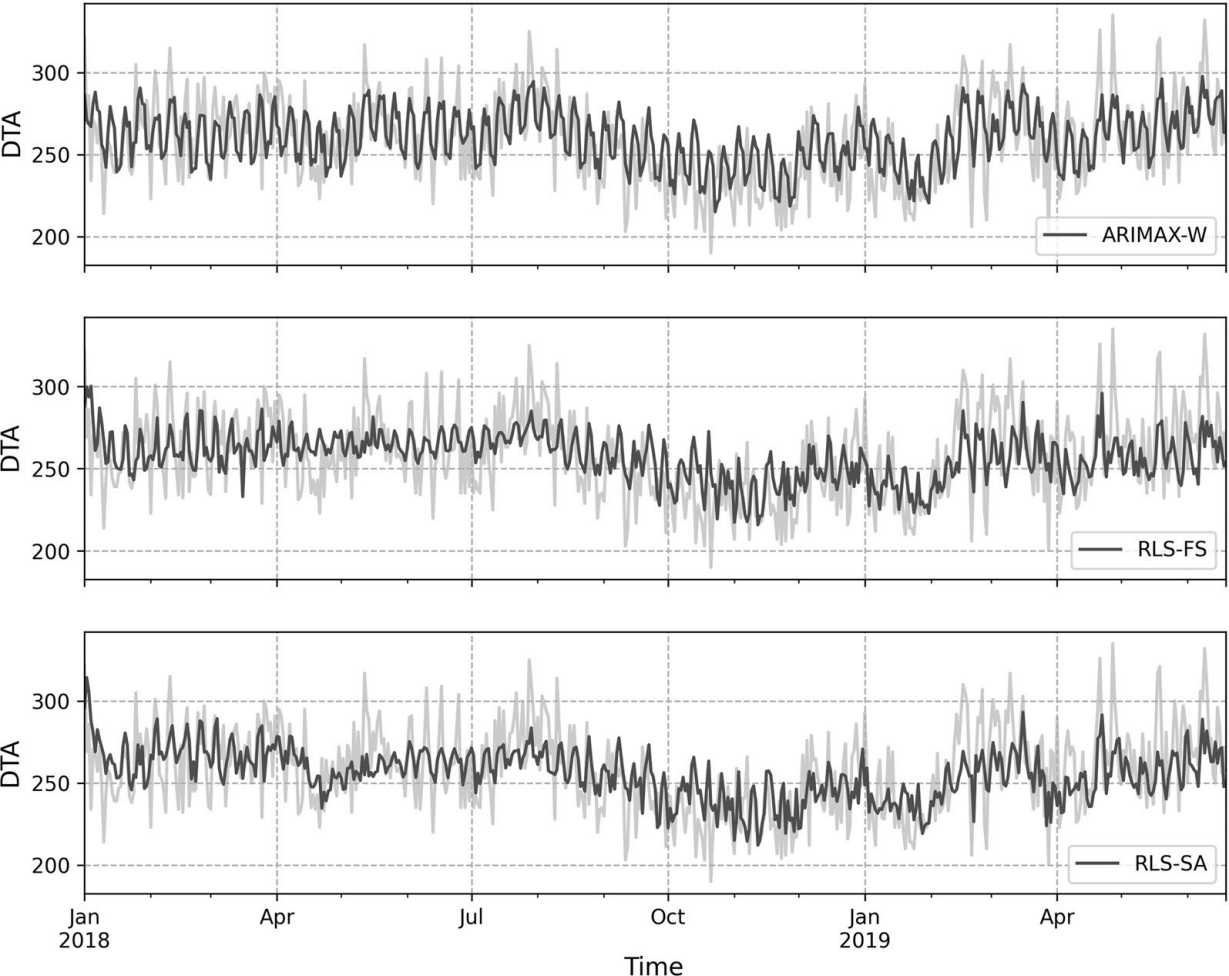
Three best performing models. Light grey line = ground truth, dark grey line = prediction. ARIMAX-W = regression with ARIMA errors using features identified by Whitt et al. [13], RLS = recursive least squares, SA = simulated annealing, FS = floating search

ANOVA showed statistically significant differences between models with *p* < 0.001. Model performance and multiple comparisons are presented in Table 2 and predictions are visualized in Figs. 2 and 3. ARIMAX-W(2, 0, 2) provided the best out-of-sample accuracy with MAPE of 6.6% but did not differ statistically from RLS-FS or RLS-SA. Estimated coefficients of this model are provided in Table 3. RLS was identified as the second-best model with MAPE of 6.9% when trained with SA features and MAPE of 6.9% when trained with features identified by FS. Univariate LMS resulted in MAPE of 7.0%. LMS-U, RLS-SA and RLS-FS outperformed univariate ARIMA(1, 0, 0)x(1, 0, 0)_7_ which provided an accuracy of 7.1%. Predictions of the models compared to ground truth are shown in Fig. 2 and Fig. 3. Detailed residual analysis of the model performance is provided in the digital supplementary materials (Additional File 3).

**Table 2:** Model accuracies in terms of absolute percentage errors

	Mean	Standard deviation	Median	Max	Differs from SN (*p*)	Worse than best (*p*)
Naive	8.4	6.4	6.9	36.4	1.00	** < 0.001**
ARIMAX-A	8.4	6.2	6.9	33.7	1.00	** < 0.001**
RLS-U	8.3	6.2	7.1	37.7	1.00	** < 0.001**
SNaive	8.2	6.6	6.6	41.8		** < 0.001**
ARIMAX-SA	8.0	6.5	6.5	39.0	1.00	** < 0.001**
RF-FS	8.0	5.9	6.6	33.5	1.00	**0.002**
LMS-FS	7.8	5.9	6.5	32.6	0.98	**0.007**
RF-SA	7.7	5.7	6.5	28.5	0.72	**0.035**
RF-U	7.5	5.7	6.1	33.2	0.42	0.10
RF-A	7.4	5.7	6.4	36.6	0.22	0.22
LMS-A	7.3	5.6	6.3	34.3	0.16	0.30
ARIMAX-FS	7.3	5.9	5.9	36.2	0.12	0.37
LMS-SA	7.2	5.5	6.1	31.6	0.07	0.53
RLS-A	7.2	5.5	6.4	39.3	**0.048**	0.64
ARIMA	7.1	5.5	5.7	29.5	**0.019**	0.86
LMS-U	7.0	5.3	5.8	30.7	**0.011**	0.95
RLS-SA	6.9	5.1	5.9	24.6	**0.003**	1.00
RLS-FS	6.9	5.2	5.9	30.1	**0.002**	1.00
ARIMAX-W	6.6	5.3	5.3	31.7	** < 0.001**	

*ARIMA* autoregressive integrated moving average, *ARIMAX* regression with ARIMA errors, *RLS* recursive least squares, *RF* random forest, *LMS* least mean squares, *SA* simulated annealing, *FS* floating search, SNaive = seasonal naïve, *A* all features, *U* univariate, *W* Whitt’s features. Statistical significance is calculated using two-tailed ANOVA with Dunnet’s post hoc test for multiple comparisons

**Table 3:** Estimated coefficients of the ARIMAX-W(2, 0, 2) model

	Estimate	Standard error	*p*
January	112.93	3.68	< 0.001
February	111.17	3.30	< 0.001
March	101.35	3.80	< 0.001
April	90.24	3.70	< 0.001
May	83.41	4.70	< 0.001
June	84.78	3.49	< 0.001
July	81.19	4.08	< 0.001
August	78.43	4.39	< 0.001
September	86.49	3.69	< 0.001
October	88.64	3.46	< 0.001
November	94.97	3.09	< 0.001
December	109.51	3.16	< 0.001
Monday	170.97	2.00	< 0.001
Tuesday	148.29	1.94	< 0.001
Wednesday	147.47	1.97	< 0.001
Thursday	145.46	2.23	< 0.001
Friday	164.24	2.04	< 0.001
Saturday	176.05	2.10	< 0.001
Sunday	170.63	2.05	< 0.001
Min temp	0.45	0.21	0.03
Max temp	0.89	0.23	< 0.001
Holiday + 1	5.68	3.35	0.09
Holiday + 0	− 8.57	2.99	< 0.001
Holiday − 1	19.12	2.66	< 0.001
φ_1_	− 0.11	0.14	0.44
φ_1_	0.69	0.10	< 0.001
θ_1_	0.28	0.14	0.05
θ_2_	− 0.58	0.10	< 0.001
σ^2^	352.37	16.26	< 0.001

*ɸ* non-seasonal autoregression, *θ* non-seasonal moving average

**Fig. 4:**
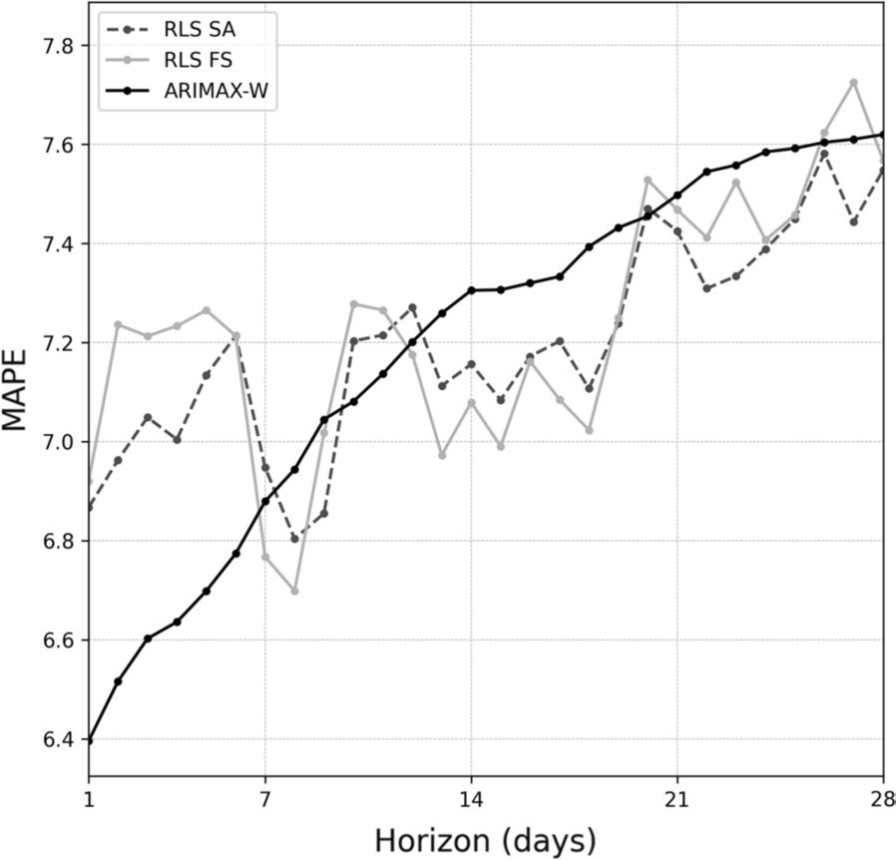
Error as function of predictive horizon for the three best performing models. ARIMAX-W = regression with ARIMA errors using features identified by Whitt et al. [13], RLS = recursive least squares, SA = simulated annealing, FS = floating serach, MAPE = mean absolute percentage error

Additionally, multi-step accuracy of the three best performing models was investigated for each predictive horizon up to 28 days into the future. For each model, the selected feature set was the same as in the next day prediction task. For ARIMAX-W, the multi-step forecast at time $t$ for horizon $h$ was generated in a standard manner [20] by simply running the 1-day forecast procedure $h$ times, with the predicted values of the previous horizons concatenated to the input time series and unobserved residuals set to zeros. For RLS-SA and RLS-FS, to generate the forecast for each horizon $h$ the filter was run for the test set with the prediction target at time $t$ set to the number of arrivals at time $t + h$. Error as a function of increasing predictive horizon is visualized in Fig. 4 for the three best performing models, showing gradual decline in accuracy with no statistically significant differences between the models irrespective of the predictive horizon. Detailed multi-step results for one-way ANOVA and Dunnett’s post hoc test are provided in Additional File 4.

### Identified features

For the sake of brevity, only features identified by better performing RLS are presented here. RLS-SA identified a total of 62 features, out of which 30 were individual public events, 11 were available beds vectors from wards, and 8 were holiday variables. *Ekström’s visits* were included as were the numbers of major and all public events. All weekdays were included except Saturday. *December*, *September,* and *March* were identified as impactful. Out of weather variables all but *snow depth* were excluded. Please see Table 4 for details.

**Table 4:** Most important explanatory variables for next day arrivals identified by simulated annealing and recursive least squares

Feature family	Feature
Website visits	Ekströms visits
Holiday name	Independence day eve
Holiday name	Easter day
Holiday name	Shrove sunday
Holiday name	All saint’s day
Holiday name	May day
Holiday name	Ascension day
Holiday	Holiday_t+0_
Holiday	Holiday_t+1_
Available hospital beds	Regional hospital A, Ward 9
Available hospital beds	Health centre 10
Available hospital beds	Regional hospital A, Ward 8
Available hospital beds	Health centre 12
Available hospital beds	Regional hospital A, Ward 5
Available hospital beds	Health centre 11, Ward 3
Available hospital beds	Health centre 2
Available hospital beds	Health centre 11, Ward 2
Available hospital beds	Regional hospital B, Ward 1
Available hospital beds	University hospital, ED ward
Available hospital beds	Health centre 11, Ward 1
Month	December
Month	September
Month	March
Public event	30 individual public events*
Public event	Number of major daily public events
Public event	Number of total daily public events
Weather	Snow depth
Weekday	Sunday
Weekday	Monday
Weekday	Wednesday
Weekday	Friday
Weekday	Thursday
Weekday	Tuesday

*Individual public events are not shown here due to their high number

RLS-FS identified a total of 55 features, out of which 29 were individual public events and 7 were individual holidays. Website visits to both domains were included. Out of weather variables all but *cloud count* were excluded. All weekdays were included, but out of months only *March*, *February* and *December* were considered significant. Please see Table 5 for details.

**Table 5:** Most important explanatory variables for next day arrivals identified by floating search and recursive least squares

Feature family	Feature
Holiday name	Shrove sunday
Holiday name	Easter day
Holiday name	Midsummer
Holiday name	Christmas eve
Holiday name	All Saint’s day
Holiday name	Independence day eve
Holiday name	Ascension day
Holiday	Holiday_t−1_
Available hospital beds	Health centre 2
Available hospital beds	Health centre 11, Ward 1
Available hospital beds	University hospital, ED ward
Calendar variable	Working day
Month	March
Month	February
Month	December
Public event	29 individual public events*
Public event	Number of major public events
Weather	Cloud count
Website visits	Website visits_tays.fi/acuta_
Website visits	Website visits_tays.fi_
Weekday	Thursday
Weekday	Saturday
Weekday	Friday
Weekday	Wednesday
Weekday	Tuesday
Weekday	Sunday
Weekday	Monday

*Individual public events are not shown here due to their high number

## Discussion

To the best of our knowledge, this was the first study to investigate feature selection in truly high-dimensional multivariate ED forecasting. We demonstrated that using high-dimensional multivariate input in conjunction with appropriate feature selection slightly enhances predictive accuracy when compared to using complete feature set or a univariate model. Calendar variables, load of secondary care facilities and local public events were dominant in the identified predictive features. The best predictive model achieved an absolute percentage error of 6.6 to 7.1%. The models demonstrate a similar, relatively linear decay over a horizon of 28 days.

Both feature selection methods resulted in a somewhat similar collection of features and in almost identical predictive accuracies. A high number of local public events was included in both feature sets, some of which are intuitively unlikely to have marked impact on ED service demand mostly due to their small size. It is possible that some public events end up in the final feature set not because they are especially important but simply because of their abundance. For example, in the case of FS, a high number of features increases their likelihood to appear in the addition phase which might risk an increase in false positives. It is also difficult to differentiate the impact of the weekly seasonality from the impact of the public events since most of the public events are naturally organized in the weekend. It is possible that the weekly seasonality “leaks” into the public event variables due to multicollinearity with calendar variables. Similar seasonality also likely explains the inclusion of certain calendar months in the feature sets.

Capacity of many secondary care facilities was prominent among explanatory variables identified by SA. If any underlying causality can be assumed, it serves to highlight the interdependent nature of the ED and importance of access block as an important contributor to overcrowding as previously suggested by [27] and as hypothesised above.

RLS-FS provided better accuracy than the 8.4% that was documented by Whitt et al. using a ARIMAX model [13]. However, reproducing the approach of *Whitt el al* on our data (ARIMAX-W) produced the best accuracy with 6.6% suggesting that MAPE errors are not directly comparable over different facilities despite the desired scale-invariance of the metric. *Ekström *et al. documented one day ahead accuracy of 6.1% in two ED’s with similar size as ours using a General Linear Model (GLM) with website visits and calendar variables as inputs [10]. Interestingly both of our feature selection algorithms included website visits in the final feature set supporting findings of *Ekström *et al. but, the resulting accuracy was slightly worse than they documented. We believe this is at least in part due to relatively short validation set of 3 months used by *Ektsröm *et al., in which the inability of a GLM to adjust to changes in the time series does not become evident in the manner that can be seen with RF in our study (Fig. 2) which leads to overly optimistic interpretation of model performance.

To the best of our knowledge, as previously stated, there is only one article that has previously investigated feature selection processes specifically in the context of ED forecasting by Jiang et al. [15]. They documented an approach in which a Genetic Algorithm was used for feature selection prior to fitting a Deep Neural Network (DNN). However, their initial feature space contained mere 22 dimensions consisting completely of calendar and weather variables and it begs the question of whether performing dimensionality reduction in their setting makes sense in the first place. This question will remain unanswered, since they don’t document the performance of DNN with the complete feature set. Moreover, Jiang et al. divided their test set of 128 days into 6 folds and report aggregated accuracies for different forecasting horizons. For these reasons it is impossible to make meaningful comparisons between their and our results.

In broader context, feature selection in multivariate time series forecasting is a relatively under-examined subject and readily available software solutions do not exist. For this reason, it would be interesting to see how our approach generalises into other domains such as industrial, commercial, or econometric forecasting in which high-dimensional multivariate time series are abundant but manual feature selection is either impractical or impossible. In retail, for example, the number of target variables of interest are often counted in tens of thousands, and costs of performing any manual model engineering for each target independently greatly surpasses the benefits of potential aggregated accuracy increase. However, computational extraction of relevant features as suggested in this study could result in significant accuracy increase with marginal labour cost.

Neural networks (NN) are readily applied in fields such as machine vision in which number of input dimensions is inherently extremely high, but their use specifically in time series prediction has been a challenge. Only recently a NN used in conjunction with a statistical model outperformed pure statistical time series tools in the M4 time series forecasting competition [20]. Following this result, some potentially performant multivariate NN algorithms for time series forecasting have appeared [28] and documenting their performance in ED forecasting with high number of features would be an interesting subject for a follow-up study. Alternatively, retaining the computationally lower requirements of statistical modelling, further work could entail incorporation of a feature selection step into the auto-ARIMA procedure itself. In addition to potentially better performance, this approach would have the additional benefit of bringing feature selection capabilities conveniently to the same interface already widely used by the forecasting community.

### Limitations

Despite the carefully performed cross validation and moderate size of the validation set, this was a retrospective cohort study, and its results must be confirmed in a prospective setting. This is mainly due to inherent uncertainty in the accuracy of the older visit statistics. Our study suggests that adding non-conventional exogenous variables such as public events and availability of hospital beds and operation room schedules as inputs in a predictive model might increase model performance. However, availability of these inputs in a prospective setup might be a challenge in a hospital with suboptimal IT infrastructure. We observed a significant drop in the DTA from September 3, 2018 onwards due to a reorganization of the ED in which underaged patients were redirected to a newly opened pediatric ED. This most likely has a negative impact in the model performance, and it should be considered when interpreting the results. There was a non-trivial amount of missing data in available hospital beds because the software that was used to monitor capacities was introduced sequentially one hospital at a time during the period of our train set. Missing values were imputed using constant zero. This might have had a negative impact on model performance. Please see Fig. 1 for visual representation. The list of local public events provided to us was intuitively non-exhaustive with some well-known events missing, which risks overly pessimistic evaluation of their importance. Otherwise, no missing data was observed. We also note that due to lack of available weather prediction data, historical weather variables were used instead of weather predictions.

### Conclusions

Our study provides new insight into potential underlying factors associated with number of next day presentations. It also suggests that predictive accuracy of next day arrivals can be increased using high-dimensional feature selection approach when compared to both univariate and nonfiltered high-dimensional approach. Performance over multiple horizons was similar with a gradual decline for longer horizons. However, outperforming ARIMAX remains a challenge when working with daily data. Future work should focus on enhancing the feature selection mechanism, investigating its applicability to other domains, and in identifying other potentially relevant explanatory variables.

## Supplementary Information

**Additional file 1.**Target variable data. The table contains all daily total arrivals in a machine-readable format observed in the study period (1/6/2015 – 19/6/2019). (XLS 39 kb).

**Additional file 2.**ARIMAX code. The archive contains code that was used to produce ARIMAX results. (ZIP 12 kb).

**Additional file 3.**Residual analysis. The document contains detailed residual analysis of the models performance. (DOCX 4.9 Mb).

**Additional file 4.**Statistical analysis of multi-step predictions. The spreadsheet contains statistical analysis of differences in accuracies between multi-step predictions generated with the three best performing models. (XLSX 11 kb).

## Data Availability

Complete time series of daily total arrivals is provided along with this manuscript (Additional File 1). Weather data is publicly available using an online service maintained by Finnish Meteorological Institute. Other explanatory data that support the findings of this study are available from Tampere City, Istekki Ltd and Unitary Healthcare Ltd but restrictions apply to the availability of these data, which were used under license for the current study, and so are not publicly available. Data are however potentially available from the authors upon reasonable request and with permission of Tampere City, Istekki Ltd and Unitary Healthcare Ltd.

## Abbreviations

ARIMA: Autoregressive integrated moving average
ARIMAX: Autoregressive integrated moving average with exogenous features
ARIMAX-W: ARIMAX with Whitt's features
DTA: Daily total arrivals
ED: Emergency department
FS: Floating search
GLM: General linear model
IT: Information technology
LMS: Least mean squares
MAPE: Mean absolute percentage error
RF: Random forest
RLS: Recursive least squares
SA: Simulated annealing
SARIMA: Seasonal ARIMA
Snaive: Seasonal naive
X-A: Model X with all features
X-FS: Model X with features identified using FS
X-SA: Model X with features identified using SA
X-U: Model X with univariate signal
