# Supplementary figures and images for: Forecasting daily emergency department arrivals using high-dimensional multivariate data: a feature selection approach
Source: BMC Med Inform Decis Mak. 2022 May 17;22:134. doi: 10.1186/s12911-022-01878-7 (PMC9112570; doi:10.1186/s12911-022-01878-7)

Appendix

Residual analysis


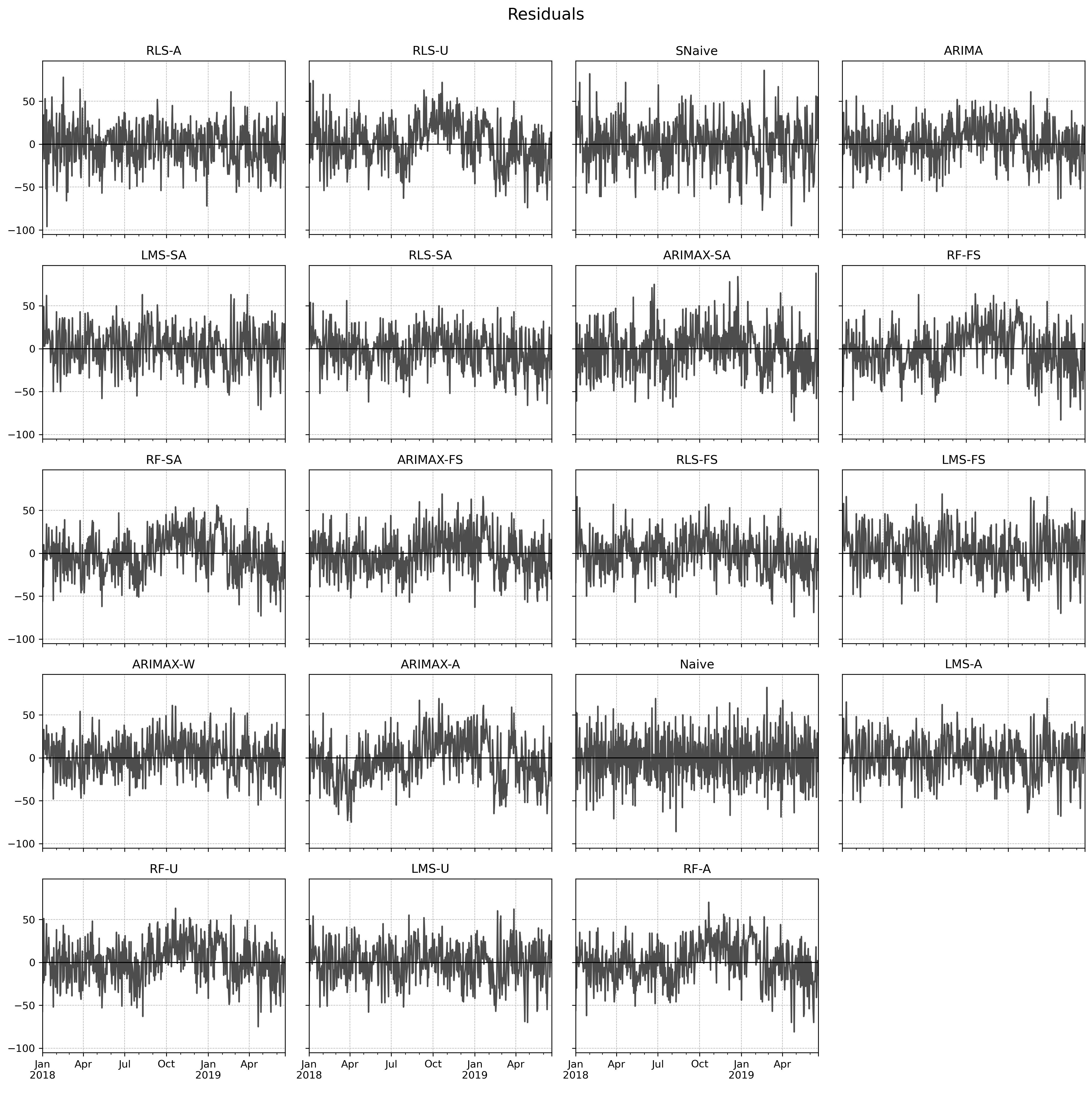


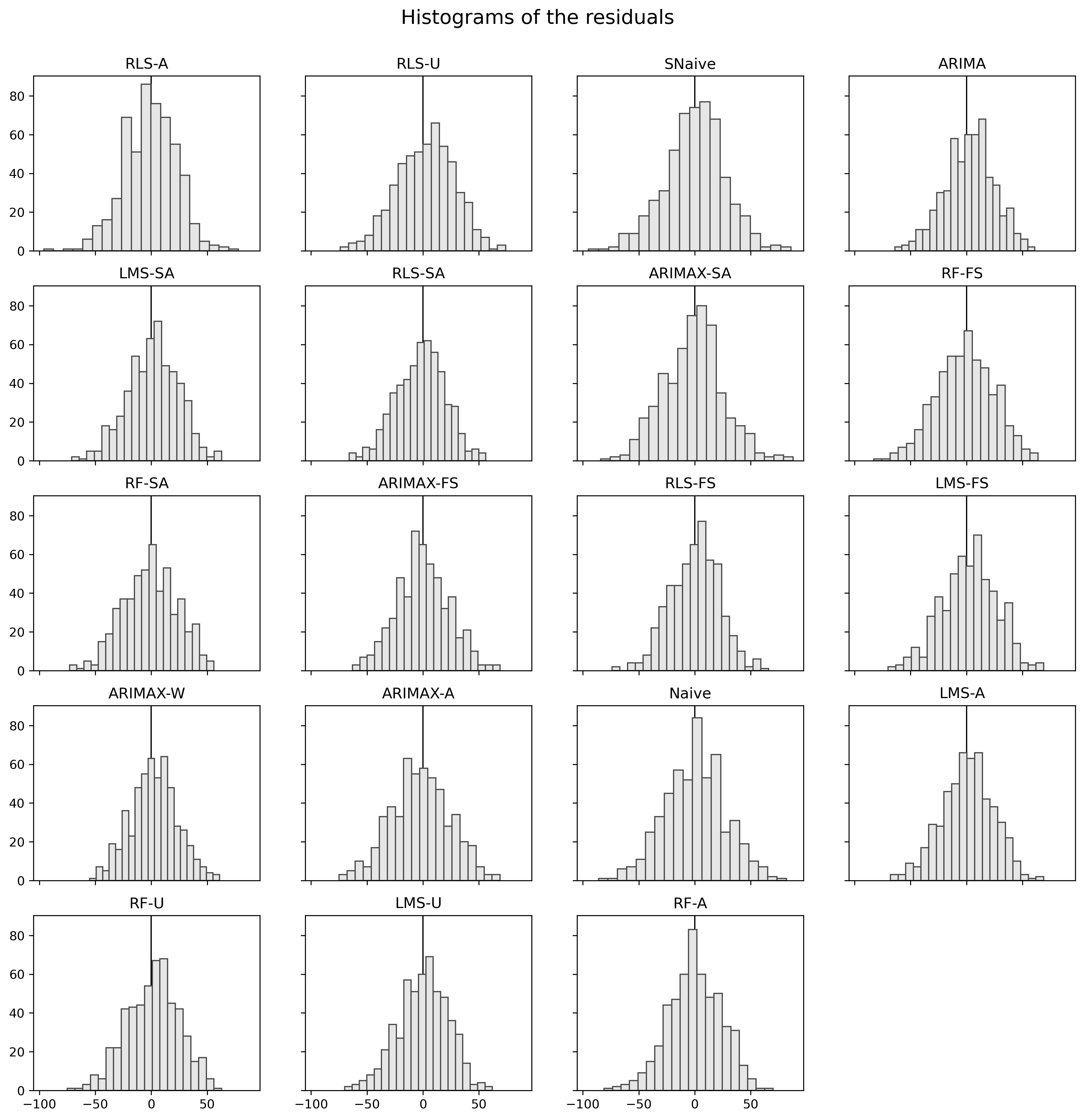


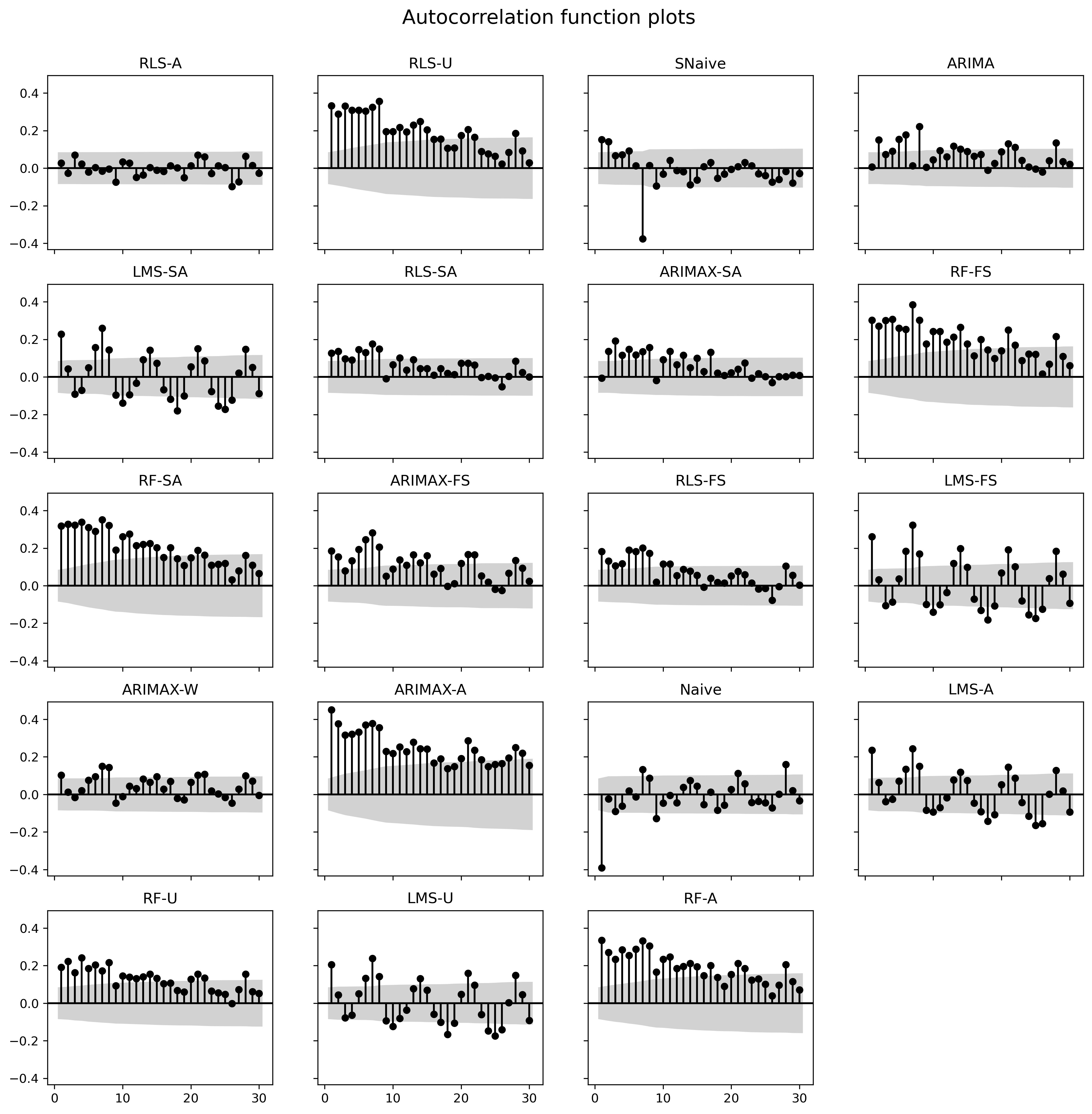

Supplement: Supplementary file 3 — Additional file 3.Residual analysis. The document contains detailed residual analysis of the models performance. (DOCX 4.9 Mb). [file 12911_2022_1878_MOESM3_ESM.docx]
